# Functional expression and ligand identification of homo- and heteromeric *Drosophila melanogaster* CO_2_ receptors in the *Xenopus laevis* oocyte system

**DOI:** 10.1371/journal.pone.0295404

**Published:** 2023-12-29

**Authors:** Paul M. Ziemba, Alina Mueck, Günter Gisselmann, Klemens F. Stoertkuhl

**Affiliations:** 1 AG Physiology of Senses, Ruhr-University Bochum, Bochum, North Rhine-Westphalia, Germany; 2 Department of Cell Physiology, Ruhr-University Bochum, Bochum, North Rhine-Westphalia, Germany; USDA Agricultural Research Service, UNITED STATES

## Abstract

Carbon dioxide (CO_2_) is an important olfactory cue in *Drosophila melanogaster* and can elicit both attractive and aversive behaviors. It is detected by gustatory receptors, Gr21a and Gr63a, found in the ab1C neuron in basiconic sensilla on the third antennal segment. Volatile substances that modulate the receptors’ function are of interest for pest control. While several substances block ab1C neurons or mimic the activating effect of carbon dioxide, it is not known if these substances are indeed ligands of the CO_2_ receptor or might act on other proteins in the receptor neuron. In this study, we used the recombinant *Xenopus laevis* expression system and two-electrode voltage-clamp technology to investigate the receptor function. We found that application of sodium bicarbonate evokes large inward currents in oocytes co-expressing Gr21a and Gr63a. The receptors most likely form hetromultimeric complexes. Homomultimeric receptors of Gr21a or Gr63a are sufficient for receptor functionality, although oocytes gave significantly lower current responses compared to the probable heteromultimeric receptor. We screened for putative blockers of the sodium bicarbonate response and confirmed that some of the substances identified by spike recordings of olfactory receptor neurons, such as 1-hexanol, are also blockers in the *Xenopus* oocyte system. We also identified a new blocking substance, citronellol, which is related to insect repellents. Many substances that activate receptor neurons were inactive in the *Xenopus* oocyte system, indicating that they may not be ligands for the receptor, but may act on other proteins. However, methyl pyruvate and *n*-hexylamine were found to be activators of the recombinant Gr21a/Gr63a receptor.

## Introduction

Carbon dioxide (CO_2_) is a colorless gas found in the Earth’s atmosphere. Its concentration in the atmosphere is currently (2022) about 0.042% [1]. However, the CO_2_ concentration can vary depending on the environment, time of day, and location. Most organisms produce CO_2_ as a byproduct of cellular respiration, while photosynthetic organisms convert it into carbohydrates via carbon fixation. The CO_2_ concentration in a local environment can influence the behavior of animals. Humans are not able to smell or taste atmospheric levels of CO_2_, but recent studies show that high concentrations above 30% (300,000 ppm) can be detected [2]. In mammalians CO_2_ sensation occurs indirectly, by an intracellular acidification caused by the diffusion of CO_2_ into the mucosal sensory neurons and its conversion into carbonic acid by an intracellular carbonic anhydrase. This activates the transient receptor potential ankyrin 1 channels (TRPA1) as recent studies have shown for the murine TRPA1 receptor [3]. A similar mechanism, involving the intracellular carbonic anhydrase CAII [4], activates the receptor guanylate cyclase GC-D [5], which triggers a cGMP-mediated signaling cascade involving the cyclic nucleotide-gated channel CNGA3 [6].

In contrast, insects have a CO_2_ detection system composed of receptors that directly accept CO_2_ as an agonist. The molecular basis of insect CO_2_ sensing receptors was first described in the model organism *Drosophila melanogaster* [7, 8]. Olfactory receptor neurons of ab1C basiconic sensilla in *Drosophila melanogaster* co-express the two gustatory receptor genes, DmelGr21a and DmelGr63a, which form most likely heteromeric receptor. Co-expression of both subunits in the neuron is necessary to maintain CO_2_ sensitivity [7, 8]. Analysis of olfactory (Or) and gustatory (Gr) insect receptors shows enough sequence homology for classification of both receptor families into one superfamily of insect chemoreceptors [9]. The number of Gr genes is expanded in other insect species. For example, the malaria vector *Anopheles gambiae* has three receptor subunits named AgamGr22-24, of which AgamGr22 and AgamGr24 are clear homologs to DmelGr21a and DmelGr63a [10]. Insect olfactory and gustatory receptors have a 7-transmembrane domain structure, with the N-terminal end located intracellularly [11, 12]. Heterologous expression studies have confirmed that gustatory and olfactory insect receptors are ligand-gated cation channels [13–15]. For at least some olfactory insect receptors [15–17] as well as gustatory insect receptors [17–19], G-protein-coupled modulation or signaling has been shown. This fact is a source of controversy regarding whether insect chemoreception relies on a dual signaling mode of metabotropic and ionotropic signal transduction [20]. The *Drosophila* Gr21a/Gr63a CO_2_ receptors have been characterized in detail using spike recordings of olfactory receptor neurons. In addition to the CO_2_ response, other chemicals that activate or inhibit the receptor-expressing neurons have been identified [21, 22]. However, these experiments used the spike rate of the CO_2_-sensitive neurons as the read-out signal in the intact fly. To our knowledge, currently, no investigations have reported the expression of the *Drosophila* receptors in recombinant systems that allow for the direct measurement of receptor-mediated CO_2_-evoked currents by electrophysiological techniques.

In the present work, we describe the functional expression of the *Drosophila* CO_2_ receptors in the *Xenopus laevis* oocyte system and its characterization by the two-electrode voltage-clamp method. This system was further used to screen for activating or blocking substances.

## Materials and methods

### *Xenopus laevis* oocyte expression

The cDNAs coding for the *Drosophila melanogaster* receptors Gr21a or Gr63a were cloned into the expression vector pcDNA3 using standard PCR methods. cRNAs were prepared using the Ampli Cap-Max^™^ T7 High Yield Message Maker Kit (CellScript, Inc.) following the manufacturer’s protocol. *Xenopus laevis* oocytes were kindly provided by the laboratory of Prof. Dr. M. Hollmann in the department of biochemistry I—receptor biochemistry of the Ruhr-University Bochum, Germany. As previously described, oocytes were obtained from female *Xenopus laevis* frogs that were anesthetized with 0.06% (w/v) ethyl-2-aminobenzoic acid (methansulfonate salt; Sigma-Aldrich, Taufkirchen, Germany) for 30 minutes [23]. Ovary tissue was removed and placed in Barth’s solution (88 mM NaCl_2_, 1 mM KCl, 0.82 MgSO_4_, 0.33 mM Ca(NO_3_)_2_, 0.42 mM CaCl_2_, 2.4 mM NaHCO_3_, 5 mM Tris-HCl, pH 7.4, 100 U/ml penicillin, and 50 μg/ml streptomycin). Afterwards, oocytes were treated with collagenase (2 mg/ml Type II) in Ca^2+^-free Barth’s solution for 1.5–2 h at room temperature. Healthy stadium IV-VI oocytes were selected for cytoplasmic cRNA injection. Each oocyte was injected with 5–20 ng of receptor coding cRNA using the nanoliter injector 2000 (WPI, Berlin, Germany). Injected oocytes were placed in fresh Barth’s solution and incubated at 18 °C. Oocytes were measured 2–4 days after the injection.

### Electrophysiology

Electrophysiological recordings were performed as described [24]. Agonist-induced currents were recorded using the voltage-clamp mode of the two-electrode voltage-clamp technique (Turbo Tec-3X npi, Tamm, Germany). The holding potential was -60 mV unless stated otherwise. Voltage-clamp electrodes were pulled from borosilicate glass capillaries (1.17 x 1.50 x 100 mm, Science Products, Hofheim, Germany), and the electrode resistance was 0.5–1.5 MΩ when filled with 3 M KCl. During the electrophysiological measurements, oocytes were continuously bathed in normal frog Ringer’s solution (NFR) as follows: 115 mM NaCl, 2.5 mM KCl, 1.8 mM CaCl_2_, 10 mM HEPES, pH 7.2. Sodium bicarbonate solutions were dissolved directly in NFR prior to all experiments and subsequently, the pH was adjusted with HCl. Carbonated NFR was prepared by aerating NFR with CO_2_ for 30 min. These freshly prepared solutions were used within 5 min for the experiments. Stock solutions of different chemicals (0.1 or 1 M) were made in dimethyl sulfoxide (DMSO). Substances were further diluted in NFR to the stated concentration. Drugs were delivered to the oocyte (100 μl) using an automatic pipette (research pro, Eppendorf, Germany). Currents were recorded using the Pulse 8.4 software (HEKA, Ludwigshafen, Germany) or CellWorks 6.1.1 (NPI, Tamm, Germany).

### Data analysis

Currents were measured using the acquisition software mentioned above. To monitor changes in the agonist-induced currents, a standard concentration was applied after 1–5 test substances were applied. To consider time-dependent sensitization or desensitization of receptors, amplitudes were normalized to the mean amplitude of the standard application before and after the test substance application. Concentration-response data for agonist-evoked currents were fit with the Hill equation using SigmaPlot 8.0. Data are expressed as the standard error of the mean (S.E.M.). Data sets were tested for statistically significant differences using Student’s t-test from SigmaPlot 8.0, and were corrected for multiple parallel tests using the Bonferroni method. Significance is indicated by * (p<0.05) or ** (p<0.01).

### Chemicals

All chemicals were purchased from Sigma-Aldrich (Germany) unless otherwise stated.

## Results

### Functional expression of *Drosophila* CO_2_ receptors

We first investigated the functionality of the *Drosophila* CO_2_ receptors in the *Xenopus laevis* oocyte expression system. Recently, it was reported that the homologous receptors of the moth *Helicoverpa armigera* can be activated by sodium bicarbonate when expressed in the *Xenopus* system [25]. Therefore, we applied different concentrations of sodium bicarbonate dissolved in normal frog Ringer`s solution (NFR) as well as NFR carbonated with CO_2_ to oocytes expressing Gr21a and Gr63a alone or the combination of both.

**Fig 1:**
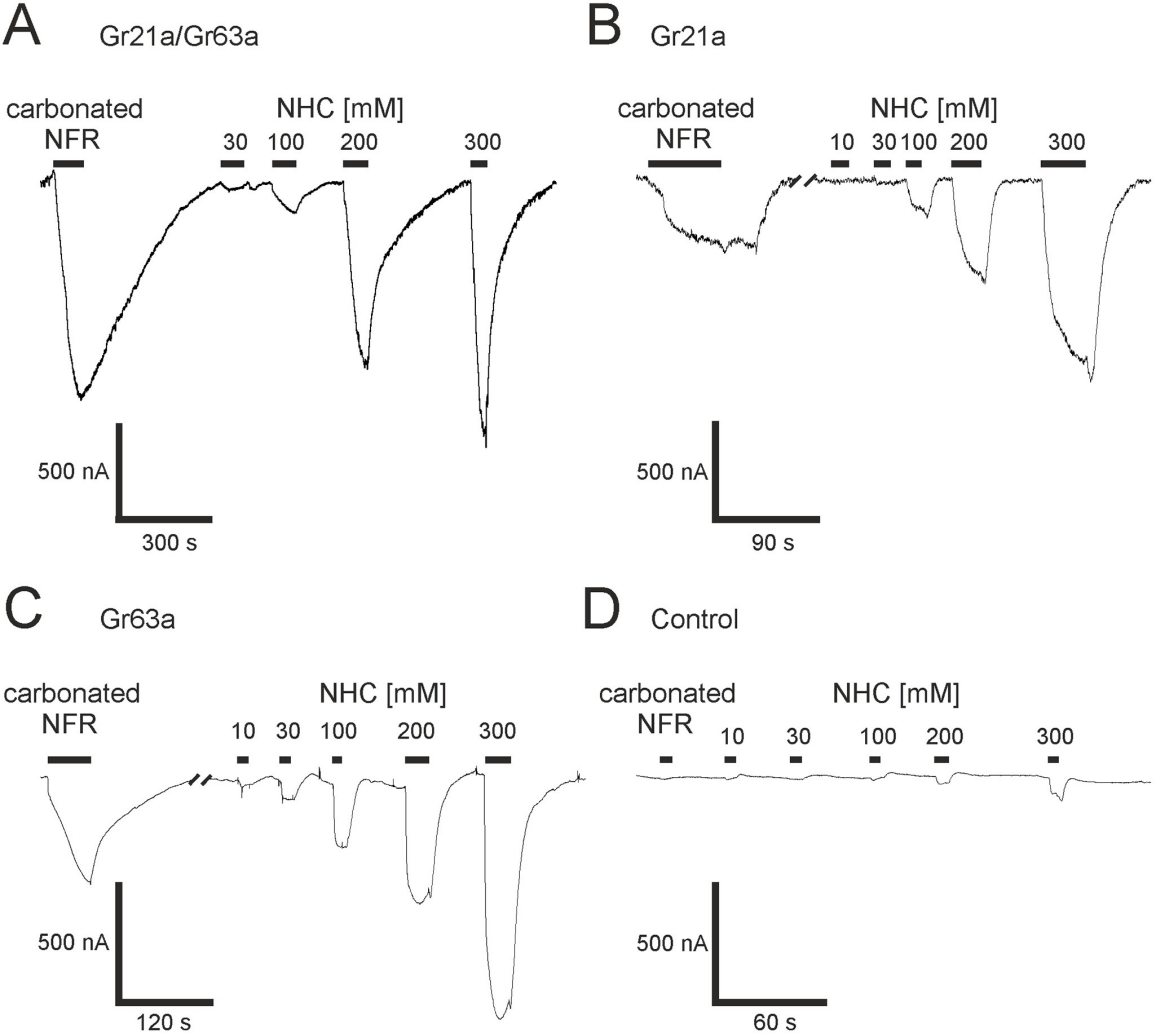
Activation of the *Drosophila* CO_2_ receptors in the *Xenopus* expression system. Original registration of Gr21a/Gr63a (A), Gr21a (B), and Gr63a (C) receptor-mediated currents induced by carbonated NFR or different concentrations of sodium bicarbonate (NHC). (D) Control measurement with non-injected oocytes. Black bars indicate the duration of the application.

In oocytes co-expressing the combination of Gr21a/Gr63a, the application of sodium bicarbonate generated currents in a dose-dependent manner, reaching amplitudes of 2.9 ± 1.0 μA (n = 8–9) for Gr21a/Gr63a which were significantly larger than in non-injected oocytes (p<0.05) (Fig 1A–1D). Sodium bicarbonate evoked smaller currents in oocytes expression Gr21a (1.9 ± 0.6 μA, n = 8) or Gr63a (2.5 ± 1.2 μA, n = 6) which were also significantly different from non-injected oocytes as controls (0.176 ± 0.043 μA, n = 7–15) (Fig 1B–1D). To ensure that Gr21a/Gr63a is not activated by the elevated sodium concentration due to the sodium bicarbonate application, we applied NFR supplied with 300 mM sodium gluconate as a control. The current evoked by these control applications was significantly smaller than those by sodium bicarbonate. This proves that the activation is caused by bicarbonate and not by sodium ions or elevated osmolarity (S1 Fig).

Carbonated NFR was also an agonist and elicited currents of 3.0 ± 1.4 μA (n = 6) for Gr21a/Gr63a and 0.3 ± 0.06 μA (n = 6) for Gr21a or 0.2 ± 0.05 μA (n = 5) for Gr63a alone. We compared this response to the current evoked by 300 mM bicarbonate, which was the highest concentration we could apply. Carbonated NFR elicited 87 ± 13% of the bicarbonate response in oocytes expressing Gr21a/Gr63a, 34 ± 8% (n = 6) for Gr21a (n = 6) and 28 ± 8% (n = 5) for Gr63a alone (S2 Fig).

**Fig 2:**
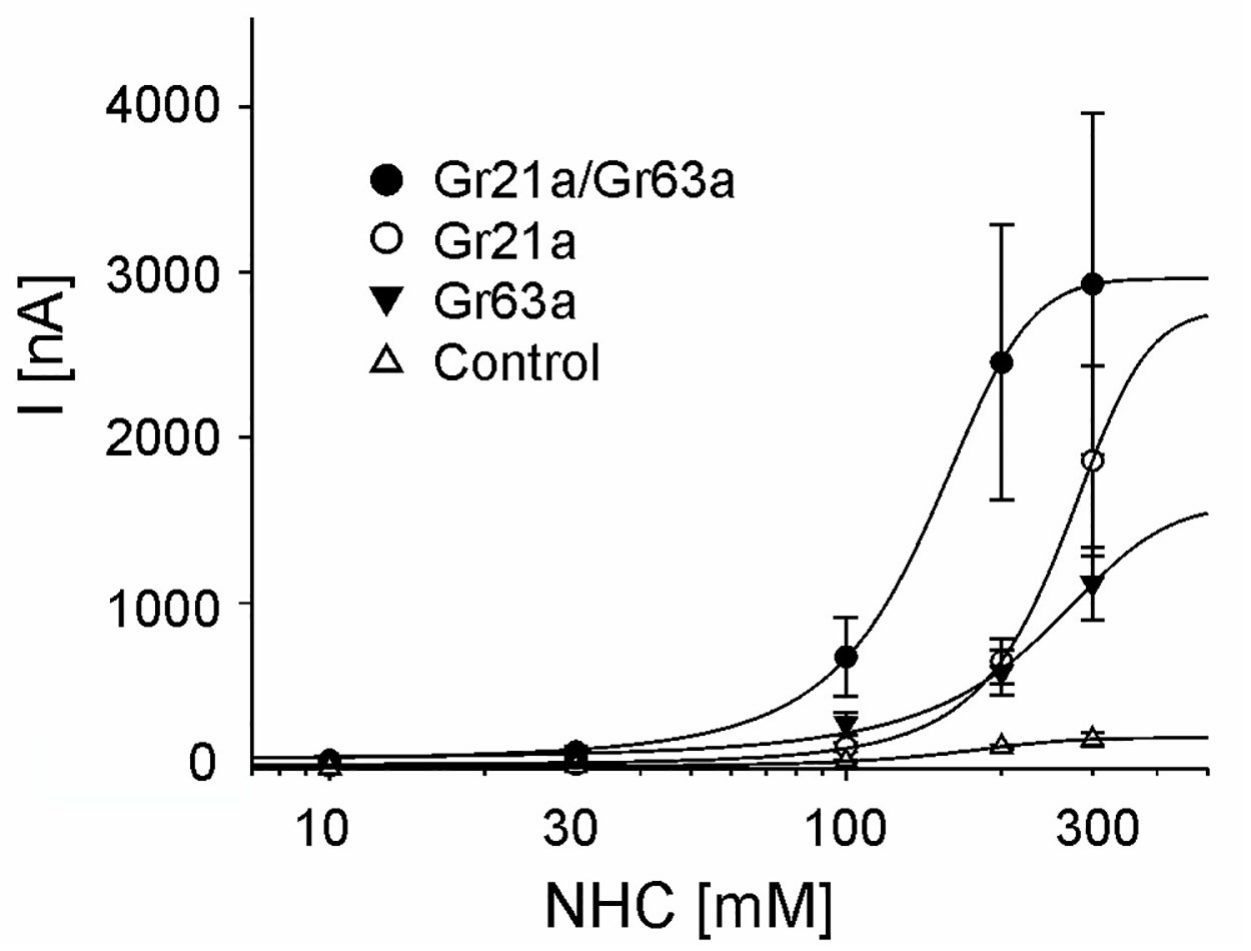
Concentration-response curves of the *Drosophila* CO_2_ receptors in the *Xenopus* expression system. Dose-dependent currents elicited by sodium bicarbonate (NHC) in oocytes expressing CO_2_ receptors. (Gr21a: n = 8, Gr63a: n = 5–6, Gr21a/Gr63a: n = 7–11, non-injected control oocytes: n = 8–11 for each data point).

Next, we established concentration-response curves for the receptor activation by sodium bicarbonate. Gr21a/Gr63a had an EC_50_ of 142 ± 23 mM (Fig 2). Gr21a or Gr63a alone were less sensitive, however, the EC_50_ was not determined because we couldn’t apply a sodium bicarbonate concentration that gave nearly saturating current responses.

### pH-dependent activation of Gr21a/Gr63a

**Fig 3:**
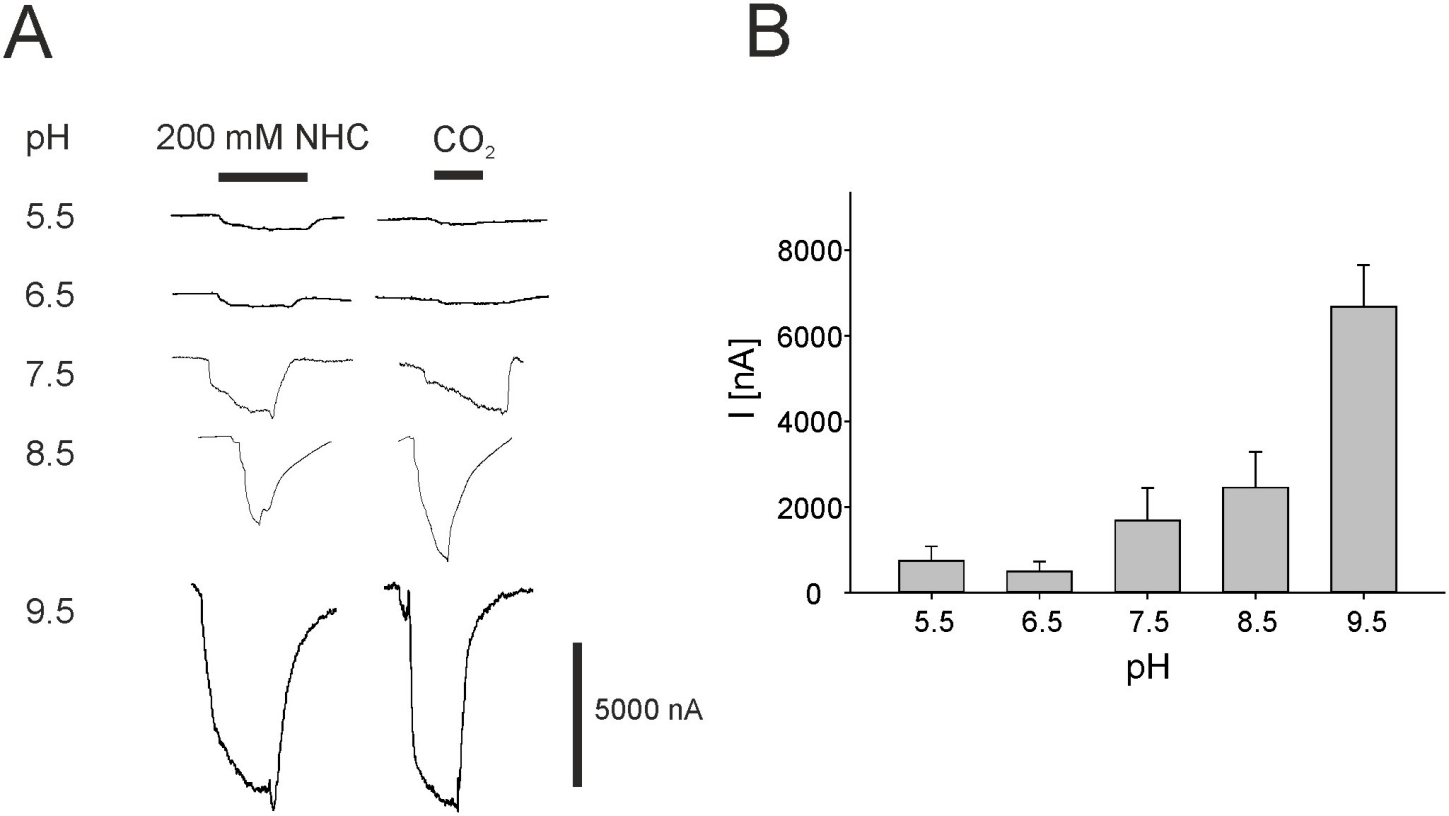
Effects of pH condition dependent response of the *Drosophila* CO_2_ receptors. (A) Original recordings of currents effected by different pH-conditions elicited by 200 mM sodium bicarbonate (NHC) or carbonated NFR in oocytes expressing Gr21a/Gr63a. (B) Current evoked by 200 mM sodium bicarbonate at the indicated pH values (pH 5.5, n = 5–6; pH 6.5, n = 5; pH 7.5 n = 8; pH 8.5, n = 8–11, pH 9.5, n = 6). Black bars indicate the duration of the application.

To address the question whether the receptor activation is affected by effects of pH conditions, we applied sodium bicarbonate in NFR as well as carbonated NFR adjusted to pH values ranging from pH 5.5 up to a pH of 9.5 to oocytes expressing Gr21a/Gr63a. The amplitude of the current response was strongly increased for sodium bicarbonate (Fig 3A and 3B) and for carbonated NFR (S3 Fig) at higher pH values. While the amplitude at pH 6.5 was slightly decreased in comparison to pH 5.5, pH values of 7.5 and higher resulted in noticeably increased responses to both sodium bicarbonate and carbonated NFR.

### Characterization of receptor ligands

**Fig 4:**
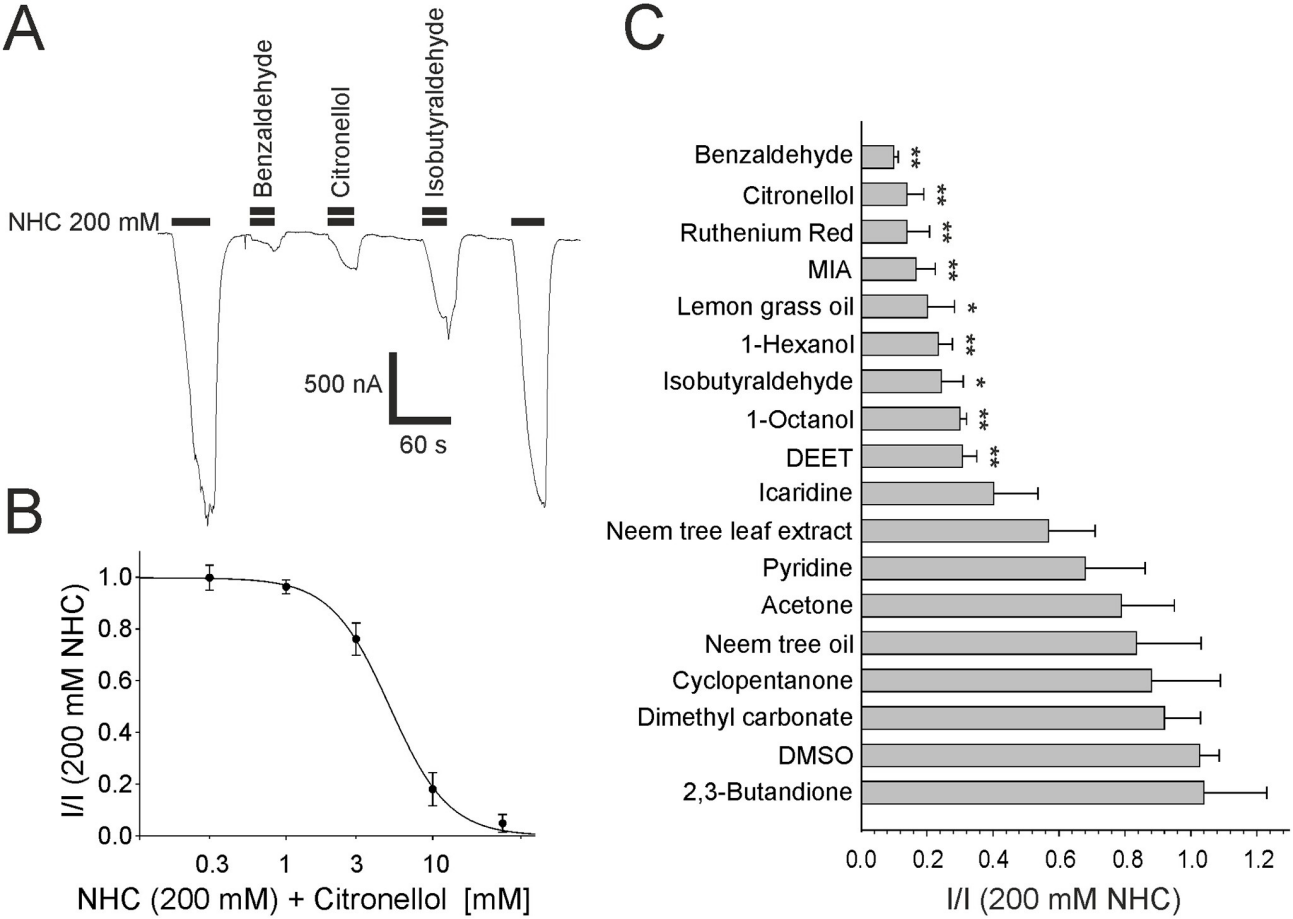
Inhibitors of the *Drosophila* Gr21a/Gr63a CO_2_ receptor. (A) Original registrations of currents elicited by 200 mM sodium bicarbonate (NHC) in oocytes expressing Gr21a/Gr63a in the presence of 10 mM of the indicated test substance co-applied with sodium bicarbonate. (B) Concentration-inhibition curve for citronellol constructed from measurements depicted in (A), n = 7–8. (C) Antagonistic action of various substances co-applied with 200 mM sodium bicarbonate. Used concentrations were 10 mM except for DEET (1 mM), MIA and Ruthenium Red (0.1 mM), and plant oils (1:100). Black bars indicate the duration of the application. Significant block is indicated by * (p<0.05) or ** (p<0.01).

**Fig 5:**
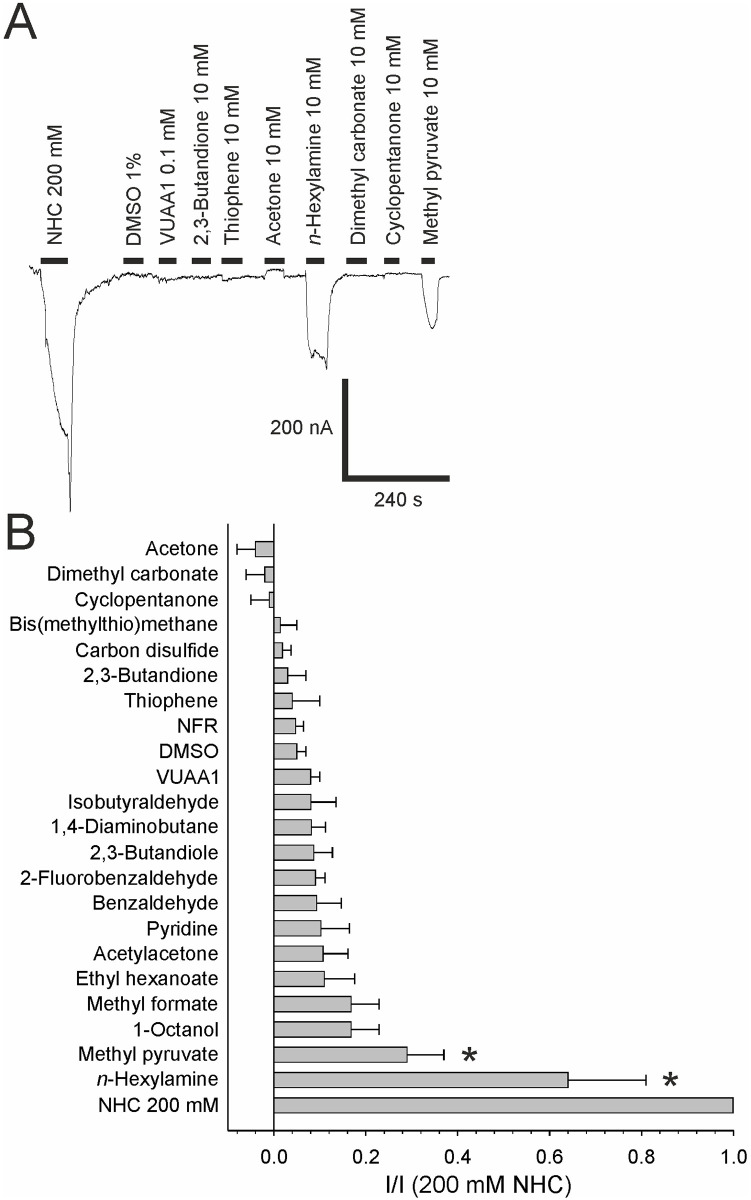
Screening of potential agonists of the *Drosophila* Gr21a/Gr63a CO_2_ receptor. (A) Original registrations of currents elicited by 200 mM sodium bicarbonate (NHC) or 10 mM of the indicated substances in oocytes expressing Gr21a/Gr63a. (B) Agonistic action of various substances normalized to the effect of 200 mM sodium bicarbonate. Used concentrations were 10 mM for each substance except for DMSO (1%) and VUAA1 (0.1 mM).

A variety of blockers or activators of CO_2_-sensitive sensory neurons were identified in *Drosophila*, *Aedes* and *Anopheles* [21, 22, 26]. It is not known if these chemicals are true ligands for Gr21a/Gr63a or act on other receptors in the receptor neuron, and thus depend on the cellular context. To clarify whether these chemicals act on Gr21a/Gr63a in a system different from insect neuronal cells, we tested some of these compounds on receptors expressed in the *Xenopus* oocyte system. In addition, we intended to screen for new ligands of the receptor. A concentration of 1% DMSO had no effect on sodium bicarbonate responses or on control oocytes (Figs 4C and 5B).

#### Antagonists screening

For blocker experiments, we used the chemicals in NRF with 200 mM sodium bicarbonate and found that some substances at 10 mM concentration can reversible reduce the sodium bicarbonate evoked response (Fig 4). Gr21/Gr63 were significantly inhibited by 1-octanol, isobutyraldehyde, 1-hexanol, lemon grass oil, ruthenium red, citronellol, and benzaldehyde which blocked the Gr21a/Gr63a receptors up to 90% (listed in increasing efficacy). In this context, citronellol showed an IC_50_ of 5.1 ± 0.25 mM (n = 8). A screening of known general blockers of insect odorant receptors like amiloride derivatives [24] revealed MIA (5-(*N*-methyl-*N*-isobutyl)amiloride) that also blocks Gr21a/Gr63a, thus with a lower effectivity compared to odorant receptors. The insect repellent DEET (*N*,*N*-diethyl-*m*-toluamide) showed only a partial block of the bicarbonate-evoked response at 1 mM concentrations (S4 Fig). DMSO, dimethyl carbonate, cyclopentanone, neem tree oil, acetone, pyridine, neem tree leaf extract, icaridin and 2,3-butadione failed to block Gr21a/Gr63a receptors.

#### Agonist screening

**Fig 6:**
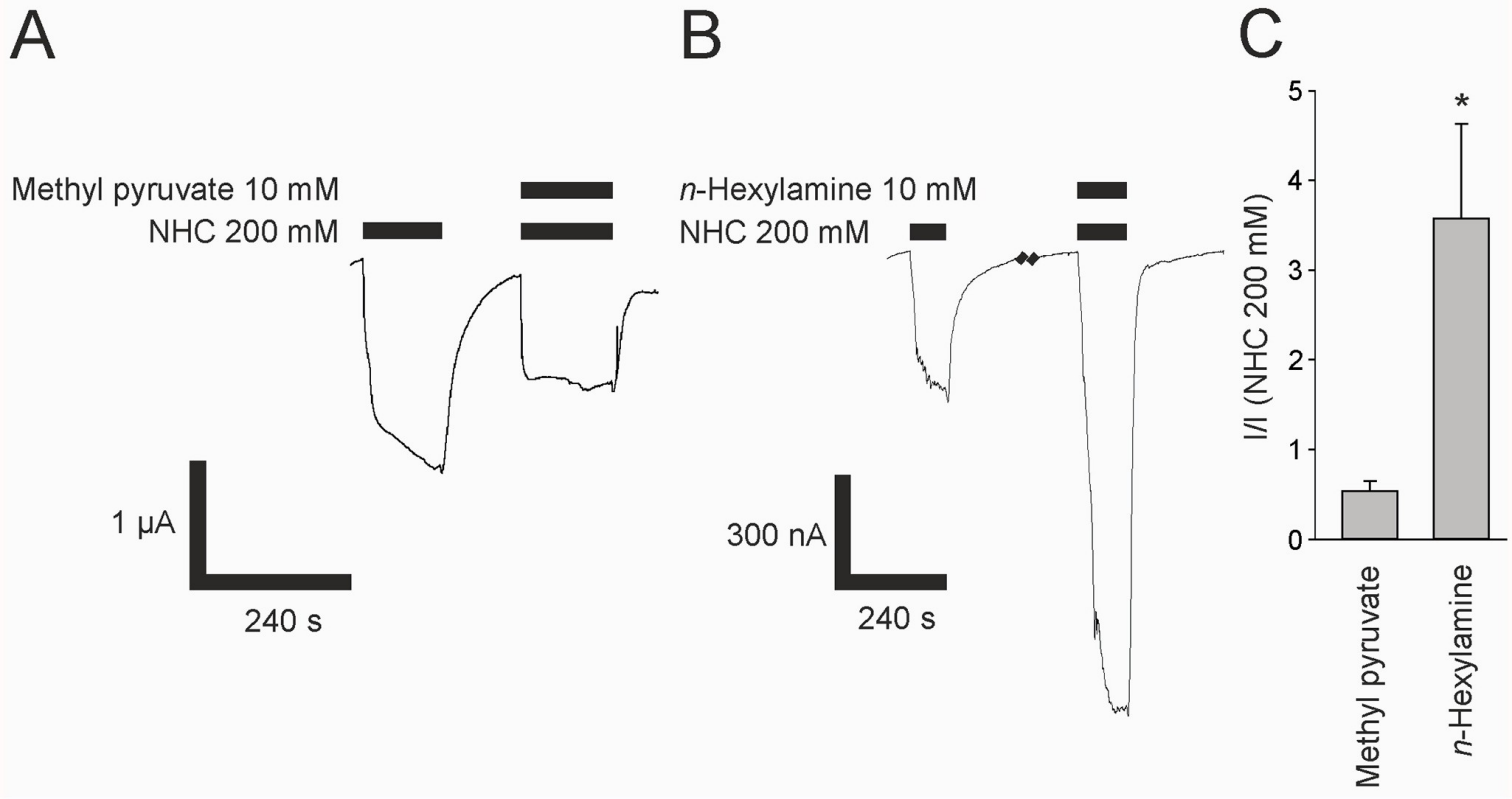
Co-application of agonists with carbonated NFR on *Drosophila* Gr21a/Gr63a CO_2_ receptor. (A) Original registration of currents elicited by 200 mM sodium bicarbonate (NHC) in oocytes expressing Gr21a/Gr63a in the presence of methyl pyruvate (10 mM) co-applied with 200 mM sodium bicarbonate. (B) 10 mM *n*-hexylamine potentiates the NHC response in oocytes expressing Gr21a/Gr63a. Black bars indicate the duration of the application. (C) Responses of co-application of 200 mM sodium bicarbonate with methyl pyruvate or *n*-hexylamine normalized to the effect of 200 mM sodium bicarbonate. Co-application of carbonated NFR together with methyl pyruvate (10 mM) evoked responses of 53±12% and together with *n*-hexylamine (10 mM) responses of 357±106% compared to the effect of 200 mM sodium bicarbonate (n = 5). Significance is indicated by * (p<0.05).

Besides CO_2_, other volatile odorants are known to induce spike activity in CO_2_ responsive insect receptor neurons [21, 22, 26]. We screened several substances for activation of Gr21a/Gr63a receptors in the *Xenopus* oocyte system at 10 mM concentrations. None of the tested substances which show activating responses in olfactory receptor neuron spike recordings, were active in the oocyte system (acetylacetone, pyridine, benzaldehyde, isobutyraldehyde, cyclopentanone, dimethyl carbonate, and acetone) [22, 26–30]. However, we identified two agonists that were previously described to block *Drosophila* and *Aedes agypti* CO_2_ evoked spike activity of olfactory receptor neurons [22, 26, 27] (Fig 5A and 5B). Methyl pyruvate at 10 mM concentration evoked currents reaching 29 ± 8% (n = 8) of the currents evoked by 200 mM NHC. Additionally, *n*-hexylamine at 10 mM concentration evoked currents reaching 64 ± 17% (n = 8) of the 200 mM NHC response. Furthermore, when applied together with 200 mM NHC, it potentiated the NHC response 3.57 ± 1.06 fold (n = 5) (Fig 6B and 6C), though methyl pyruvate failed to potentiate the NHC response (Fig 6A and 6C).

## Discussion

In our current work, we demonstrate that the *Drosophila* CO_2_ receptors Gr21a, Gr63a and the complex of Gr21a/Gr63a can be functionally expressed in the recombinant *Xenopus laevis* oocyte system. Application of bicarbonate solutions or carbonated NFR activates strong receptor-mediated cation currents when the oocytes were measured in the two-electrode voltage-clamp configuration. This is similar to what has been recently reported for homologous CO_2_ receptors in *Anopheles*, *Aedes*, and *Helicoverpa* [25, 31]. We used sodium bicarbonate solutions to activate the CO_2_ receptors in a concentration dependent manner. The presumptive heteromeric receptor DmelGr21a/DmelGr63a was somewhat less sensitive, with an EC_50_ of 142 ± 23 mM, compared to the receptors HarmGr1-3 from *Helicoverpa* (EC_50_ = 32 mM). These three subunits, HarmGr1, HarmGr2, and HarmGr3 cooperate to sense CO_2_ [25]. In addition to bicarbonate, the *Drosophila* Gr21a/Gr63a receptor can be activated by carbonated NFR, suggesting that CO_2_ could be the activating ligand as recently proposed for the receptors from *Culex quinquefasciatu*s [31]. The CO_2_ receptor shares pharmacological blockers with other gustatory and olfactory receptors. For this reason we assumed that MIA could act as an antagonist for Gr21a/Gr63a receptors. The amiloride derivative MIA, which is a blocker for insect olfactory receptors [24, 32], also blocks the CO_2_ receptors, providing a valuable tool for receptor characterization. The same is true for ruthenium red, a blocker for many Ca^2+^ channels and also for insect olfactory and gustatory receptors [14, 33].

In our experiments, we observed a clear effect dependent of pH-conditions of the CO_2_- or NHC-evoked response. For both types of activation, sodium bicarbonate solution or carbonated NFR, the current amplitude was higher at elevated pH. Dissolved CO_2_ forms an equilibrium with bicarbonate in water, and acidification of an NHC solution increases the concentration of dissolved CO_2_. Xu *et al*. observed an increase in the current amplitude for *Culex quinquefasciatus* CO_2_ receptors expressed in *Xenopus* oocytes when the NHC solution was acidified compared to neutral pH levels, suggesting that CO_2_, rather than bicarbonate, activates the receptor [31]. We observed a similar effect when comparing the amplitude evoked by NHC at pH 5.5 to that at pH 6.5. However, we noted a significantly more pronounced increase at higher, basic pH values. Under these pH conditions, virtually no dissolved CO_2_ is present in the NHC solution. At this stage of experimental data, making a definitive determination regarding the nature of the ligand is challenging. There is a strong possibility that the CO_2_ binding site is located intracellularly, as described for another type of mammalian CO_2_ receptor involved in chemical senses, the GC-D [34]. In this case, intracellular pH would be crucial for the CO_2_/HCO_3_^-^ equilibrium. Since the precise mechanism of receptor activation remains unknown, it is challenging to speculate on pH-dependent modulatory processes, as observed in other ion channels. However, the Orco receptor from the moth *Cydia pomonella* (CpomOrco) also shows a similar pH-dependency when activated by VUAA1 [35]. This behavior can be attributed to the presence of the glutamine residue Q_471_, which is conserved in the Lepidoptera group and located in the third intracellular loop. A mutation to histidine, which is conserved in Diptera at this position (e.g. in *Drosophila*), abolishes this behavior. Interestingly, in the corresponding intracellular loop of Gr21a/Gr63a, a conserved glutamine is present.

To our surprise Gr21a and Gr63a were also functional as homomeres, however, when co-expressed in *Xenopus* oocytes, *Drosophila* Gr21a and Gr63a showed increased sensitivity to CO_2_ as compared to the solitary expression of the receptors in oocytes. This raises questions about the role of subunits in ligand binding, ion pore formation, and the general requirement of all subunit types for a functional receptor. Our results demonstrate that both subunits can be activated by bicarbonate. In the recombinant SF9 cell system, receptors from *Helicoverpa armigera* (HarmGr1 and HarmGr3) have been shown to function as heteromultimers in a calcium imaging assay [36]. However, in this study HarmGr3, the homolog of DmelGr63a, was activated also as homomer by sodium bicarbonate. The DmelGr21a homolog, HarmGr1 could not be stimulated with CO_2_ when solitary expressed. In *Drosophila* olfactory receptor neurons, co-expression of both receptor subunits is necessary to maintain the responsiveness of the neuron to CO_2_, as investigated in the empty neuron system [7, 8].

In *Drosophila*, the CO_2_ response of receptive neurons can be blocked by several chemically non-related substances, and volatile odorant chemicals can also activate CO_2_-sensitive neurons [21, 22, 26]. The actions of blockers and activators are partly conserved between different insect species, such as in *Aedes* and *Anopheles* [26–28, 30]. The response pattern classifies CO_2_-sensitive neurons as broad range polymodal sensors, however, it is not known if this due to the polymodal nature of the CO_2_ receptor protein or mediated by additional chemoreceptors in the cell. The recombinant *Xenopus* oocyte expression system provides a good opportunity to determine whether these chemicals are ligands for the Gr proteins or not. We tested a selection of the most promising potential receptor ligands, covering substances of several chemical classes.

Several substances significantly reduced the sodium bicarbonate-evoked current in *Xenopus* oocytes, including 1-hexanol. These results are consistent with their described block of the CO_2_ response obtained from spike recordings of olfactory receptor neurons [21, 22]. These substances also block the CO_2_ responses in other insect species [21, 22, 26–28, 30]. This suggests that *Drosophila*, *Aedes*, *Culex*, and *Anopheles* receptors have at least partially conserved pharmacology for blockers. Additionally, we identified a new blocker, citronellol, a potential insect repellent [37], although it was only active at higher concentrations.

In contrast, the proposed activators cyclopentanone, acetone, thiophene, pyridine, carbon disulfide (CS_2_), and dimethyl carbonate [22, 26–29, 38] did not activate the receptor in the *Xenopus* system. Benzaldehyde, a proposed activator in *Drosophila* and *Anopheles* [22, 26], was instead a blocker in the *Xenopus* system. In the recombinant *Xenopus* system, all tested potential activators failed to evoke a current response that was significantly different from the application of control solutions in Gr21a/Gr63a-expressing oocytes. This is an astonishing result as these substances increase the spike activity in CO_2_-sensitive neurons in different insect species. In some cases, there are indications that these chemicals might act on other receptors in the cells. Chemicals like cyclohexanone are activators of the CO_2_-responsive cpA neuron housed in the maxilary palp of *Anopheles gambiae* [28]. However, cyclohexanone failed to activate when *Anopheles* Gr22, Gr23, and Gr24 are co-expressed in the *Drosophila* empty neuron system [28]. This is a clear indication that cyclohexanone is not a ligand for the CO_2_ receptor, but may act on other proteins in the cpA neuron.

This fits with the findings that a general co-expression of other members of insect chemoreceptor classes is observed in CO_2_-sensitive neurons, namely chemo-sensitive ionotropic glutamate receptors (Irs) and the Orco co-receptor for Ors. In *Aedes agypti*, the ligand-selective receptor Ir75g and the co-receptor Ir25a are found in the Gr1-3 expressing A-neuron [39]. In *Drosophila*, Gr21a/Gr63a are co-expressed with Ir25a and, in a subpopulation of cells, also with Orco [40]. However, in *Drosophila*, no ligand-selective counterparts for Ir25a or Orco were identified in the respective neurons. Another explanation for the failure to detect currents in the *Xenopus* system might be that ligands other than CO_2_ act on other receptor proteins in olfactory receptor neurons. This could subsequently activate Gr21a/Gr63a by metabotropic signaling, analogous to the phospholipase C-mediated mechanism proposed for other Gr proteins [19]. However, the existence of such signaling mechanisms for CO_2_ receptors is still elusive.

To our surprise, *n*-hexylamine and methyl pyruvate, which are known blockers of CO_2_-evoked spike activity in recordings of olfactory receptor neurons, were found to be activators of the recombinant receptor. Neuronal activity is monitored through single sensillum recording, using spike activity as a readout signal. However, this method only allows for an indirect conclusion regarding receptor activation, as it involves the participation of many other ion channels and signaling proteins in spike generation. It is possible that these activating ligands lead to a rapid desensitization of the neuronal response, thereby giving the appearance of a block in the CO_2_ response. In the case of *Aedes aegypti*, *n*-hexylamine has been shown to activate the CO_2_-sensitive glomerulus 1 when investigated using Ca-imaging techniques [41]. However, this activation persists in *Aedes aegypti* Gr3 mutants, suggesting that it is independent of a functional *Aedes* CO_2_ receptor. In contrast to this, our results demonstrate that *n*-hexylamine can act as a ligand for the recombinant CO_2_ receptor, at least in *Drosophila*.

## Conclusion

In conclusion, we have demonstrated the functionality of both probable homo- and heteromultimeric *Drosophila* Gr21a and Gr63a receptor proteins in the *Xenopus laevis* oocyte expression system. Our findings show that both subunits alone can form functional receptors, although with a lower sensitivity for CO_2_, providing important insights into the relative importance of different receptor subunits. Our results indicate that Gr21a alone is sufficient for CO_2_ activation. Screening of potential activators of the Gr21a/Gr63a receptor revealed that the tested substances may not be ligands for the receptor protein, but could generate spike activity of the olfactory receptor neurons through other mechanisms. However, we were able to confirm that some of the blockers identified by data obtained from spike activity of olfactory receptor neurons, such as 1-hexanol, are also active in the *Xenopus* system, indicating that they are indeed ligands for the receptor. Investigation of potential activators revealed pronounced differences between the recombinant CO_2_ receptor protein and the response pattern of the spike activity CO_2_-receptive olfactory receptor neurons. Thus, additional receptors could be reason for the polymodal response pattern. In the future, the *Xenopus* expression system provides a valuable tool for screening and identifying new ligands for these receptors, and for clarification of the exact role of Gr21a/Gr63a in the context of the polymodal chemoreceptive neurons.

## Supporting information

S1 Fig Application of sodium gluconate in NFR to *Drosophila* CO_2_ receptors. (A) Original registration of Gr21a/Gr63a receptor-mediated currents induced by various concentrations of sodium gluconate (NGN) in NFR or by 300 mM sodium bicarbonate (NHC). (B) Control measurement with non-injected oocytes. Black bars indicate the duration of the application. (C) Currents evoked by 300 mM NGN in Gr-expressing oocytes in relation to the NHC response.
(TIF)

S2 Fig Normalized activation of the *Drosophila* CO_2_ receptors by carbonated NFR. In oocytes expressing Gr21a/Gr63a or Gr21a, carbonated NFR elicited 87±13% (Gr21a/Gr63a, n = 6), 34±8% (Gr21a, n = 6) or 28±8% (Gr63a, n = 5) of the response evoked by 300 mM sodium bicarbonate. Significance is indicated by * (p<0.05) or ** (p<0.01).
(TIF)

S3 Fig pH-dependent activation of the *Drosophila* CO_2_ receptors by carbonated NFR. (A) Carbonated NFR elicited pH-dependent currents in oocytes expressing GR21a/Gr63a (n = 5–6 for each pH). (B) Response relative to the current evoked by 300 mM sodium bicarbonate at the respective pH.
(TIF)

S4 Fig Inhibitors of the *Drosophila* Gr21a/Gr63a CO_2_ receptor. (A) Original registrations of currents elicited by 200 mM sodium bicarbonate (NHC) in oocytes expressing Gr21a/Gr63a in the presence of MIA, DEED or RR in the indicated concentration co-applied with 200 mM sodium bicarbonate. MIA and DEET blocked reversibly. The response to sodium bicarbonate remained reduced after RR application. (B) Response like in (A) normalized to the current evoked by 200 mM sodium bicarbonate in NFR (n = 5–13). Black bars indicate the duration of the application. Significant block is indicated by ** (p<0.01).
(TIF)
