# Supplementary figures and images for: Functional expression and ligand identification of homo- and heteromeric Drosophila melanogaster CO2 receptors in the Xenopus laevis oocyte system
Source: PLoS One. 2023 Dec 29;18(12):e0295404. doi: 10.1371/journal.pone.0295404 (PMC10756536; doi:10.1371/journal.pone.0295404)

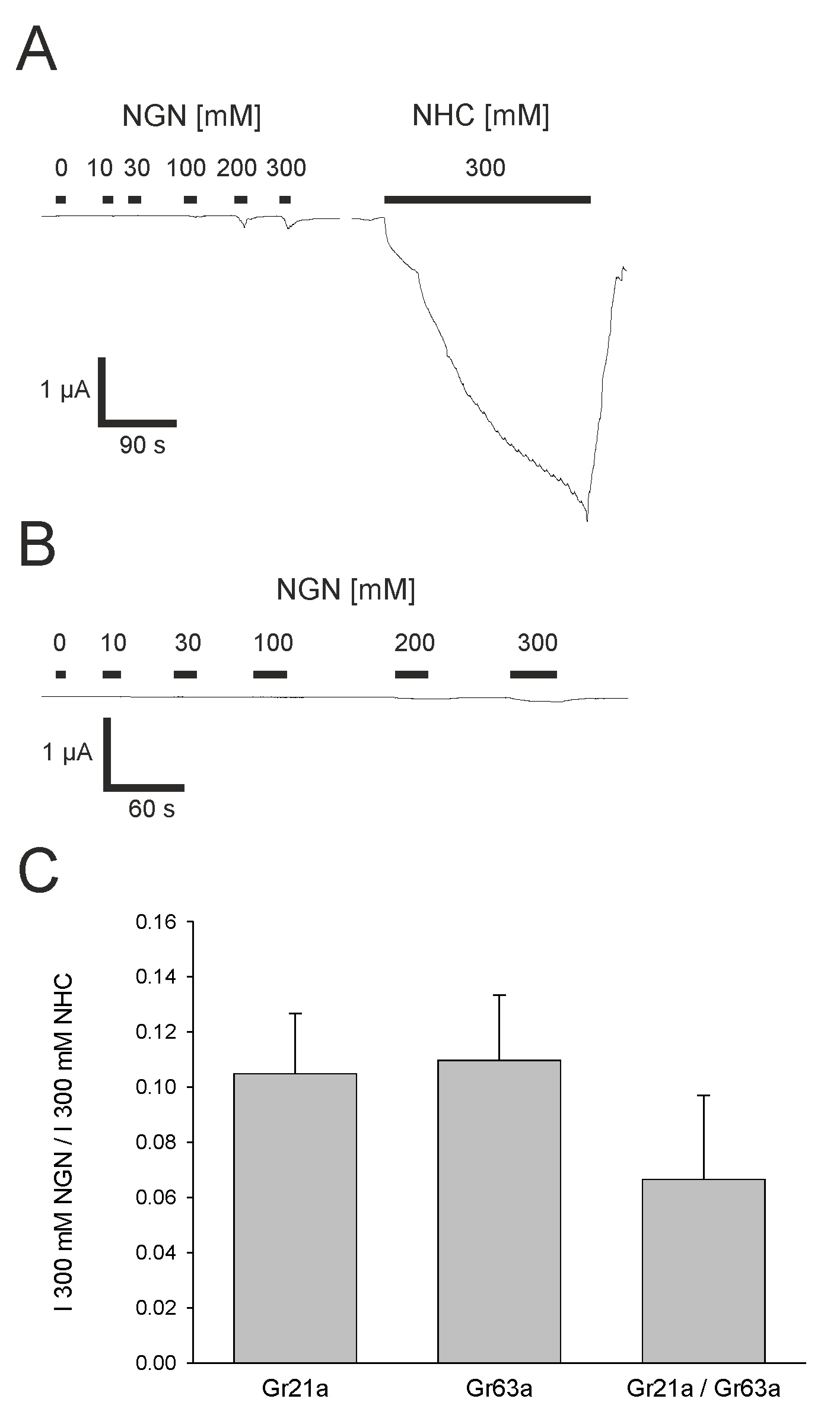

Supplement: S1 Fig — (A) Original registration of Gr21a/Gr63a receptor-mediated currents induced by various concentrations of sodium gluconate (NGN) in NFR or by 300 mM sodium bicarbonate (NHC). (B) Control measurement with non-injected oocytes. Black bars indicate the duration of the application. (C) Currents evoked by 300 mM NGN in Gr-expressing oocytes in relation to the NHC response. (TIF) [file pone.0295404.s001.tif]

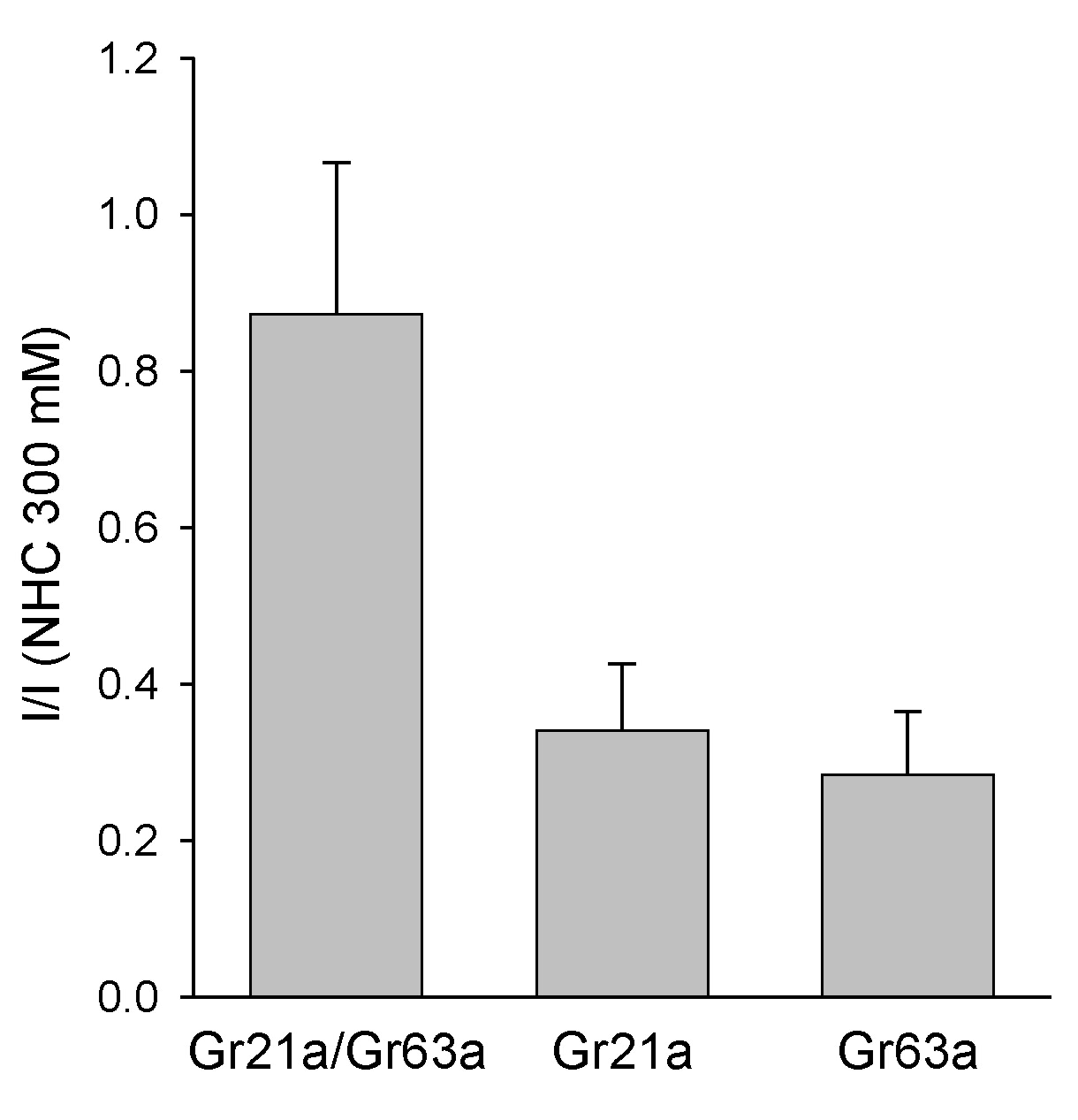

Supplement: S2 Fig — In oocytes expressing Gr21a/Gr63a or Gr21a, carbonated NFR elicited 87±13% (Gr21a/Gr63a, n = 6), 34±8% (Gr21a, n = 6) or 28±8% (Gr63a, n = 5) of the response evoked by 300 mM sodium bicarbonate. Significance is indicated by * (p<0.05) or ** (p<0.01). (TIF) [file pone.0295404.s002.tif]

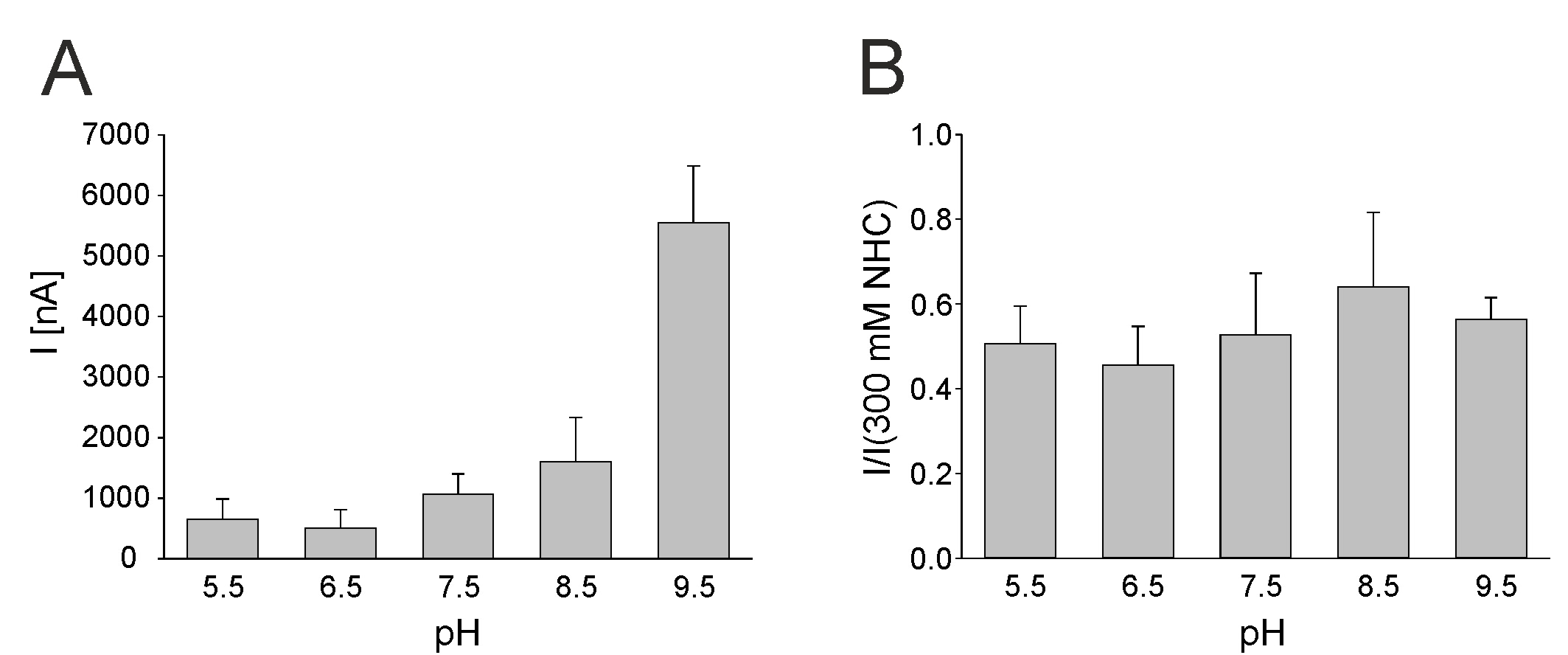

Supplement: S3 Fig — (A) Carbonated NFR elicited pH-dependent currents in oocytes expressing GR21a/Gr63a (n = 5–6 for each pH). (B) Response relative to the current evoked by 300 mM sodium bicarbonate at the respective pH. (TIF) [file pone.0295404.s003.tif]

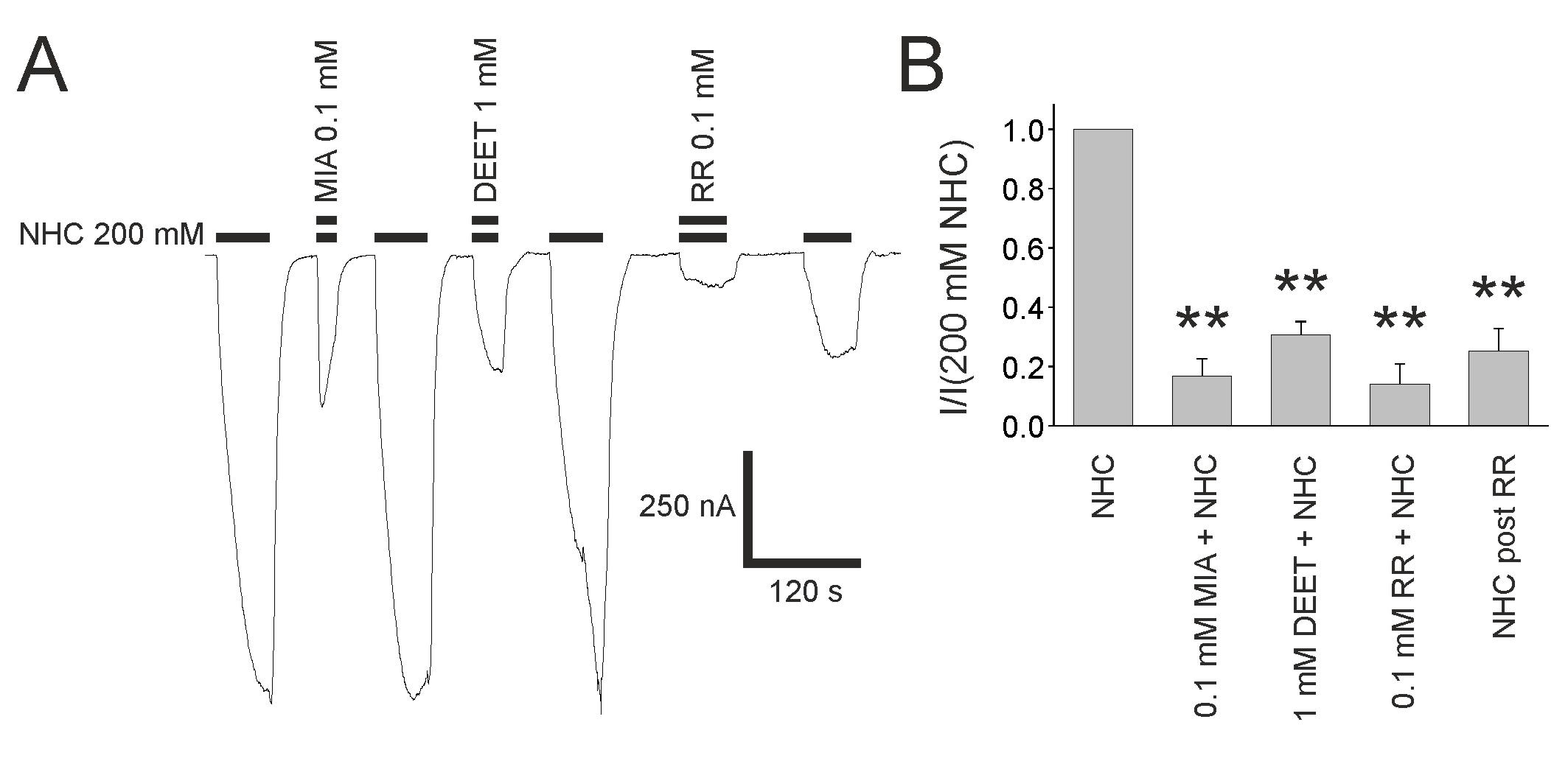

Supplement: S4 Fig — (A) Original registrations of currents elicited by 200 mM sodium bicarbonate (NHC) in oocytes expressing Gr21a/Gr63a in the presence of MIA, DEED or RR in the indicated concentration co-applied with 200 mM sodium bicarbonate. MIA and DEET blocked reversibly. The response to sodium bicarbonate remained reduced after RR application. (B) Response like in (A) normalized to the current evoked by 200 mM sodium bicarbonate in NFR (n = 5–13). Black bars indicate the duration of the application. Significant block is indicated by ** (p<0.01). (TIF) [file pone.0295404.s004.tif]
